# Cellular Mechanisms of Oxidative Stress and Action in Melanoma

**DOI:** 10.1155/2015/481782

**Published:** 2015-05-06

**Authors:** Mario Venza, Maria Visalli, Concetta Beninati, Giuseppe Valerio De Gaetano, Diana Teti, Isabella Venza

**Affiliations:** ^1^Department of Experimental Specialized Medical and Surgical and Odontostomatology Sciences, University of Messina, 98125 Messina, Italy; ^2^Department of Clinical and Experimental Medicine, University of Messina, 98125 Messina, Italy; ^3^Department of Pediatric, Gynecological, Microbiological and Biomedical Sciences, University of Messina, 98125 Messina, Italy

## Abstract

Most melanomas occur on the skin, but a small percentage of these life-threatening cancers affect other parts of the body, such as the eye and mucous membranes, including the mouth. Given that most melanomas are caused by ultraviolet radiation (UV) exposure, close attention has been paid to the impact of oxidative stress on these tumors. The possibility that key epigenetic enzymes cannot act on a DNA altered by oxidative stress has opened new perspectives. Therefore, much attention has been paid to the alteration of DNA methylation by oxidative stress. We review the current evidence about (i) the role of oxidative stress in melanoma initiation and progression; (ii) the mechanisms by which ROS influence the DNA methylation pattern of transformed melanocytes; (iii) the transformative potential of oxidative stress-induced changes in global and/or local gene methylation and expression; (iv) the employment of this epimutation as a biomarker for melanoma diagnosis, prognosis, and drug resistance evaluation; (v) the impact of this new knowledge in clinical practice for melanoma treatment.

## 1. Introduction

Reactive oxygen species (ROS), including superoxide (O_2_^−^), hydrogen peroxide (H_2_O_2_), and the hydroxyl radical (OH), are produced not only by specialised phagocytic cells, but also during normal oxidative metabolism and proliferative processes. The intracellular reduction of ROS is physiologically catalyzed by superoxide dismutase, catalase, and glutathione peroxidase (GPx) [1]. Superoxide dismutases (i.e., SOD1 and SOD2) catalyze the dismutation of the superoxide anion (O_2_^−^) to H_2_O_2_ [2, 3]. H_2_O_2_ in turn is decomposed into H_2_O and O_2_ by catalase [1], while GPx reduces lipid hydroperoxides to their corresponding alcohols and free hydrogen peroxide to water by employing glutathione (GSH) as its oxidation substrate [3].

H_2_O_2_ can even mimic the G1 to S phase transition induced by the exposure to growth factors [4, 5]. Moreover, low ROS levels may behave as second messengers of signal transduction pathways involved in cell growth, transformation, and apoptosis [6]. There are many discrepancies about the role that ROS have in the regulation of cell proliferation and the mechanisms leading to their formation. While high levels of ROS can cause cellular oxidative stress and induce apoptosis, low levels of superoxide and H_2_O_2_ can promote G1 → S cell cycle transition in various cell systems [4]. Indeed, high concentrations of H_2_O_2_ activate cell death through the activation of peroxidation reactions and come into equilibrium with Bcl2, an antiapoptotic member of Bcl family, which exerts antioxidant activity [7]. Such activity of Bcl2 is enhanced by several protein kinases activated by oxidative stress, including MAPKs, ERK1/2, and the JNK1 [8, 9]. The resulting phosphorylation of Bcl2 stimulates its antioxidant function in an attempt to block the apoptosis response. Moreover, it was found that H_2_O_2_ not only activates MAPK (ERK1/2) and Cdk2 but also can specifically downregulate p27, a significant inhibitor of Cdk2 and G1 → S cell cycle progression [10, 11], thus indicating a mechanism by which H_2_O_2_ can stimulate cell proliferation [12]. However, H_2_O_2_ is also able to regulate the cellular localization of p27Kip1 in transformed melanocytes, since melanoma cells overexpressing or treated with exogenous catalase exhibit a high percentage of p27Kip1 positive nuclei, as compared with melanoma cells deficient in catalase. The addition of H_2_O_2_ (0.1 *µ*M) to melanoma cells arrested in G1 by serum starvation induces proliferation and the cytoplasmic localization of p27Kip1. Therefore, it has been concluded that H_2_O_2_ scavenging prevents nuclear exportation of p27Kip1, allowing cell cycle arrest, and it has been suggested that cancer cells take advantage of their intrinsic prooxidant state to favour cytoplasmic localization of p27Kip1 [13]. The critical role of ROS levels in the progression from G1 → S phase is underlined by the observation that cells treated with the antioxidants or deprived of growth factors exhibit very low levels of ROS and remain quiescent [12]. These data show a strong relationship between ROS levels and cell cycle status.

## 2. Oxidative Imbalance and Human Diseases

Although cellular stress responses, such as the heat shock, unfolded protein, DNA damage, and oxidative stress, are an integral part of normal physiology to either ensure the cell's survival or alternatively eliminate damaged or unwanted cells, depending on a set of different factors, aberrant cellular stress responses are tightly linked to many common human diseases. Among these, it seems that diabetes is particularly sensitive to oxidative stresses. In type 1 diabetes the autoimmune response leads to the production of proinflammatory cytokines, which induce the apoptosis of *β*-cells by generating NO^•^ and ROS through the activation of NF-*κ*B [14, 15]. The role of the oxidative stress in the pathogenesis of type 2 diabetes is less defined. The most likely hypothesis, involving the endoplasmic reticulum stress induced by glucolipotoxicity [16], is the inability of the *β*-cells to secrete insulin. Several evidences are provided about a significant role of ROS generation and the stress response in neurodegenerative disorders, such as Parkinson's disease, in which they seem to be responsible for the loss of dopaminergic neurons [17, 18]. Moreover, a strong connection between oxidative stress and the formation of amyloid deposits has been demonstrated in other neurodegenerative disorders [19–22], indicating the important role for protein misfolding, aggregation, and formation of protein inclusions in these chronic diseases, such as Alzheimer's disease and Huntington's disease [23]. That there is a short circuit between the formation of amyloid deposits and oxidative stress has been long demonstrated in a variety of cell lines where  *β*-amyloid deposition caused activation of NADPH oxidase (NOX) and release of ROS [24, 25]. Strong heat shock response and Unfolded Protein Response (UPR) have been associated with myocardial infarction, and, furthermore, generation of ROS has been held responsible for mitochondrial damage [26] and apoptosis in cardiac myocytes [27]. Generation of reactive oxygen species has been shown to be heavily implicated in age-related macular degeneration (ARMD). In such a disease, oxidative imbalance and DNA damage are widened by chronic smoke and alcohol consumption. Therefore, these behavioral habits have been considered an aggravating factor for ARMD because of their ability to exacerbate the oxidative stress [28, 29].

## 3. Association between Oxidative Stress and Aberrant Proliferation

**Figure 1:**
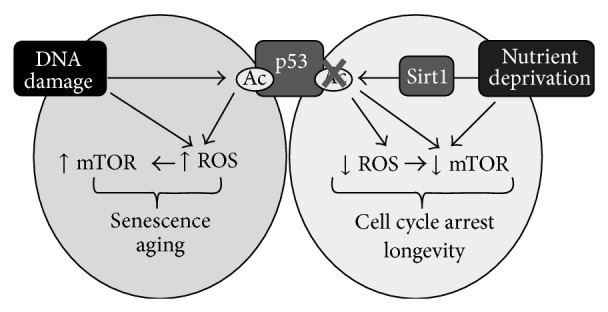
A model of how acetylation, oxidative stress, and mTOR activity might influence the response to p53. Note that this model represents an oversimplification of these signaling pathways (from [38]).

Increased expression of the protooncogene* Bcl2* or functionally activated* Bcl2* can enhance SOD, catalase, and GPx activities leading to decreased levels of ROS, retardation of G1→ S cell cycle transition, and reduced cell proliferation [3, 12, 30]. These data may lead one to deduce that braking ROS formation allows the cell to engage DNA repair processes to ensure survival, in view of increased ROS levels which may contribute to genomic instability that is a hallmark of cancer cells [12]. Indeed, although superoxide anion and other ROS have been associated for many years with oncogenesis, only recently a new role is emerging for ROS as mediators of signaling pathways leading to cell proliferation and tumor initiation and promotion. Complex and multifunctional relationships between these molecular events are being discovered which are leading researchers to believe that the tumor-promoting effects might be in relation to the tiny electric currents induced by ROS and transported through the cytoskeletal actin microfilament network [31]. As mentioned above, the regulation of ROS levels is very complex especially if one considers that ROS production is also under the control of the* TP53* suppressor gene. The induction of apoptosis by p53 has been related to its capability to induce ROS production [32]. On the other hand, ROS are also known to be critical for senescence [33] and the p53 target genes that increase ROS may also play an important role in senescence induction. However, p53 also promotes the expression of a number of antioxidant genes, accounting for p53's ability to control oxidative stress [34]. So p53's ability to decrease or increase oxidative stress likely contributes to a dual effect on senescence. Another element to be taken into account is represented by the inhibition of mTOR pathway exerted by p53 that contributes to the antisenescence activity of this transcription factor [35]. Furthermore, mTOR can be activated by ROS [36], whereby p53's antioxidant activities may reinforce the dampening of mTOR and senescence (Figure 1). Since there is good evidence that acetylation of p53 promotes senescence and apoptosis, it has been argued that inhibitors of the deacetylation enzymes might rescue p53 responses and be employed for cancer therapy [37]. The most accredited model indicates that mild stress induced by a physiological increase of cellular functions has an antioxidant response through a slight activation of p53, while a high p53 activity may induce apoptosis or senescence, thereby favoring aging. Mouse models also clearly suggest that inappropriate p53 activity promotes aging while a robust but normally regulated p53 response protects from the aging process. Therefore, the persistent stress observed in cancer would favor p53-induced senescence over a more transient cell cycle arrest [38].

Interestingly, a close interrelationship exists between oxidative stress and several stress response pathways. For example, an increase in the expression of certain inducible heat shock proteins (Hsps), particularly Hsp27, has been observed following oxidative stress [39–41]. Hsps, apart from heat shock, have been reported to protect against several oxidants. In addition, activation of the UPR stimulates upregulation of antioxidant genes through protein kinase RNA- (PKR-) like ER kinase- (PERK-) dependent phosphorylation of the Nrf2 (also known as Nfe2l2) transcription factor, whose target genes include enzymes involved in GSH biosynthesis and heme oxygenase-1 [42]. This antioxidant activity is also involved in activation of the repressor protein for Nrf2, Keap1 [43–46]. In contrast to the physiological regulation of Nrf2, in neoplasia there is evidence for increased basal activation of Nrf2. Indeed, somatic mutations that disrupt the Nrf2-Keap1 interaction, stabilize Nrf2, and activate Nrf2 target genes were found in cancer, indicating a role in tumorigenesis [47]. Interestingly, it has been shown that the Nfr2 transcription induced by endogenous oncogenic alleles of* Kras*,* Braf*, and* Myc* promotes ROS detoxification and cancer [48]. As ROS can cause damage to all of the major classes of biological macromolecules, including nucleic acids, proteins, carbohydrates, and lipids, when the cell's antioxidant defenses are overwhelmed, cell death occurs. Numerous studies have shown that the oxidative balance affects not only cell fate, but also the mode of cell death [49, 50]. Many cytotoxic agents induce ROS, including peroxide and O_2_^•−^, which are involved in the induction of apoptotic cell death [51]. H_2_O_2_ can cause the release of cytochrome* c* from mitochondria with the activation of the intrinsic pathway of apoptosis but can also activate nuclear transcription factors, like NF-*κ*B, AP-1, and p53 [52], which may upregulate death proteins or produce inhibitors of survival proteins. However, ROS are also reported to interfere with the apoptosis program, engaging cells to adopt an alternative mode of cell death. Apoptotic cell death can be switched to necrosis during oxidative stress by two possible mechanisms: inactivation of caspases or a drop in ATP levels. Caspases contain an active site cysteine nucleophile [53], which is prone to oxidation or thiol alkylation as well as S-nitrosylation [54]. This leads to their inactivation, switching the mode of cell death to necrosis [55]. However, altered redox status can promote tumor initiation and progression by blunting cell death pathways, so a prooxidant intracellular milieu has been linked to carcinogenesis and tumor promotion. To this end, increased signaling via the PI3K/Akt pathway has been shown to result in enhanced intracellular ROS generation [56]. Similarly, cancer cells that constitutively express oncogenic* Ras* have been reported to produce higher intracellular levels of O_2_^•−^ and to be resistant to drug-induced apoptosis [57]. In many tumors it has been observed that Hsps, including Hsp90, Hsp70, and Hsp27, were closely linked to the activation of tyrosine kinases, namely, Akt, and the levels of oncogenic proteins, such as Ras and HER2, strongly involved in malignancy [58, 59]. These chaperones participate in carcinogenesis and in cell death resistance by blocking key effector molecules of the apoptotic pathways at the pre- and post-mitochondrial level [59]. Thus, targeting Hsps, for example, with chemical inhibitors, is currently under investigation as an anticancer strategy [58]. Complete failure to repair DNA damage as well as inherited or acquired defects in maintenance systems of the mammalian genome can lead to mutations [60]. In addition, such deficiencies in the DNA damage response can lead to carcinogenesis, but also promotion, progression, and resistance to therapeutic treatment [60]. It is intriguing to note how some hormone-dependent cancers are strictly correlated to the types of dietary fat. A diet low in total fat, saturated, monounsaturated, and polyunsaturated fat and rich in omega-3 fatty acids, vitamin C, vitamin E, lycopene, alpha-tocopherol, selenium, beta-carotene, and quercetin is inversely associated with prostate cancer risk [61, 62]. These data highlight that the beneficial effects of antioxidant nutrients in prevention of prostate cancer derive from being able to increase the antioxidant levels.

## 4. Natural Antioxidants Prevent UV-Induced Skin Carcinogenesis

The risks of skin carcinogenesis and melanomagenesis may be lowered through the modulation of UV-activated cell signalling pathways and/or generation of oxidative stress [63]. It has been amply reported that natural antioxidants can exert a protective effect against skin cancer induced by UV radiation [64]. Medium-wave- (UVB-) induced carcinogenesis in mice was suppressed when a green tea polyphenolic fraction was topically applied to the skin or orally administered in the drinking water [65, 66]. Similarly, other reports showed that both orally administered and topically applied vitamin E [67] as well as olive oil application [68] were able to prevent the UVB-induced skin carcinogenesis in mice. Again, the anticarcinogenic effects of several antioxidants were equally well documented against melanoma. The melanomagenesis was shown to be greatly affected by substances that have the potential to inhibit ROS generation, such as genistein [69], curcumin [70], and aerial part of* P. thunbergiana* [71]. It is worth noting that genistein can act also as an “epigenetic modulator” since it can affect key tumor-related gene expression and signal pathways through dynamic regulation of both DNA methylation and histone modification pathways [72]. In this regard, D'Angelo et al. [73] indicated that hydroxytyrosol (DOPET), the major antioxidant compound present in olive oil, is able to prevent ROS production, lipid peroxidation, and protein damage in a human melanoma cell line (M14) exposed to UVA irradiation. In such a way this antioxidant exerts a protective effect on melanoma cells by reducing the UVA-induced oxidative stress.

## 5. Oxidative Stress and Epigenetic Modifications

A link, even if indirect, between oxidative stress and epigenetic alterations of either protooncogenes or tumor suppressor genes is now well established. The DNA damage caused by ROS prevents the DNA methyltransferases (DNMTs) from acting on their specific substrates, leading to global hypomethylation [74] and genomic instability. On the other hand, very high rates of ROS can reduce the expression of glutathione-s-transferase P1(GSTP1) gene, the main antioxidant enzyme, by inducing the methyl-binding protein (MBP), HDAC, and DNMT complex to methylate the promoter. High ROS levels induce also the oxidation of guanine to 8-oxoG which is able to convert the N7 position of guanine from an acceptor into a donor of hydrogen bonding, as well as to replace the 8-proton with an oxygen atom. When 8-oxoG is adjacent to the 5-methylcytosine MBP binding is weakened. Moreover, the observed frequent conversion of 5-methyl-cytosine into 5-hydroxymethyl-cytosine alters the binding affinity to MBPs resulting in epigenetic changes [75]. Generally, DNA methylation leads to gene silencing when particular and specific CpG islands are involved. In these cases the binding of transcriptional factors to their consensus sites is prevented [76]. Otherwise, the binding of methyl-binding domain proteins is favored leading to transcriptional repression through interaction with histone deacetylases (HDACs) [77, 78].

## 6. Impact of Oxidative Stress on Melanoma

Malignant melanoma, a neoplasm arising from malignant transformation of melanocytes, is predominantly a disease of the skin but may in rare instances occur at other sites, including the mucous membranes (hard palate, maxillary gingiva, lip, throat, esophagus, vulva, vagina, and perianal region) and the eye (uvea and retina). Like all tumor types there is considerable heterogeneity in outcome and molecular pathogenesis. Almost all histological and clinical patterns of melanoma are thought to be caused mainly by exposure to UV radiation, with their incidence being markedly increased in patients with a history of heavy sun exposure, or isolated episodes of serious sunburn [79]. In contrast, mucosal and soft tissue presentations of melanoma appear to have a distinct pathogenesis, as their growth might be independent of UV-linked pathways [80]. Really, the MAPK and phosphatidylinositol 3 (PI3) kinase pathways are involved differently between types or subtypes of melanoma classified according to sun exposure and anatomic site [81]. Consequently the genes concerned with these distinct pathways are differently involved, as mutations of* BRAF* [82] and* NRAS* [83] prevail in melanoma that occurs at sites intermittently exposed to UV, while a high frequency of mutations in specific exons of* KIT* is found at chronically sun-exposed or sun-protected sites, such as the mucous membranes [83]. UV can induce DNA damage through direct as well as mediated mechanisms. Mutagenic cyclobutane pyrimidine dimers, 6–4 photoproducts, DNA strand breaks, and DNA crosslinks are the direct consequences of UVB action. If not repaired properly, this DNA damage can result in mutations in the genome, ultimately contributing to skin carcinogenesis [84]. On the contrary, UVA rays are mostly responsible for DNA damage mediated by oxidative stress. However, both UVA and UVB have been shown to be responsible for photocarcinogenesis and photoimmunosuppression [85]. Epidemiological data strongly support the photoprotective role of melanin, as an inverse correlation between skin pigmentation and the incidence of sun-induced skin cancers was reported [86]. The shielding effect of melanin, especially eumelanin, is achieved by its ability to serve as a physical barrier that scatters UV radiation and an absorbent filter that reduces the penetration of UV through the epidermis [87]. DNA damage occurs to a greater extent in the upper layers of the epidermis, while the lower layers of the skin are protected as the melanin content of the skin increases [88]. Indeed, UV radiation induces less DNA damage and higher rate of apoptosis of damaged cells in darker skin than in lighter skin, a combination that results in a greatly reduced risk of carcinogenesis [88]. Another key mechanism through which UV induce melanomagenesis is the production of ROS. UV induce a dose-dependent response by human melanocytes leading to production of H_2_O_2_ [89], decrease in catalase activity, and reduced HO-1 expression [90–94]. Similarly, it has been established that there is a role of ROS in the cell damage caused by UV radiation [68, 95]. The vulnerability of melanocytes to oxidative stress can be explained by their greater ability to produce ROS compared with keratinocytes and fibroblasts due to melanin production [96]. In fact, the melanosome is thought to be the main source of the high levels of ROS observed either in melanocytes or in melanoma cells [97–102]. This hypothesis is strengthened by a higher expression of either 8-hydroxydeoxyguanosine (8-OHdG), a major form of oxidative DNA damage, or base excision repair (BER) genes in melanocytes with respect to keratinocytes [103], as well as by the decrease in ROS levels following inhibition of melanin synthesis [100]. However, there are conflicting data in the literature on the prooxidant and antioxidant effects exerted by melanin. Some studies showed that the levels of H_2_O_2_ after exposure to UV are inversely related to the amount of melanin, which would thus possess an antioxidant effect [92]. Similarly, further findings indicated that induction of melanogenesis increases the activity and expression of catalase, thus inhibiting UV-induced H_2_O_2_ generation [92, 104], and others reported that more pronounced pigmentation protects against UV- or H_2_O_2_-induced mitochondrial DNA damage [105]. In contrast, stimulation of melanogenesis is reported to promote oxidative DNA damage in human melanocytes or mouse melanoma cells [106–108].

Oxidative stress can throw off the balance of homeostasis in melanocytes, threatening their survival or inducing malignant transformation [96]. It has been reported that subunits of the NADPH oxidase (NOX) enzyme complex are strongly involved in the generation of oxidative stress and expressed in primary and metastatic melanoma cells at a higher level than in normal human melanocytes [109, 110]. In addition, NOX1 activity and protein levels increased after UV exposure in primary melanoma cells [109, 111] and may be responsible for ROS accumulation in dysplastic nevi [111]. Moreover, it was demonstrated that the expression of the neuronal form of nitric oxide synthase (nNOS) is higher in melanoma cells than in normal melanocytes [112] and that its suppression reduces xenografted melanoma tumor growth and metastatic potential in vivo [112, 113]. It is noteworthy that toxicity of reactive nitrogen species (RNS) dramatically increases in the presence of ROS [114], constituting a deleterious mix that may initiate melanomagenesis owing to the leaking of melanosome contents. The importance of oxidative stress in melanoma is reinforced by the findings that mutations in several melanoma-associated genes result from or worsen oxidative stress. For example, the somatic BRAF V600E mutation, normally occurring in nevi and melanoma, can be oxidative stress-induced [115] and loss of p16 expression, commonly observed in melanoma, leads to dramatic increases in ROS levels in cultured human melanocytes [116]. Moreover, melanoma progression is associated with depletion of PTEN and the resulting increase in superoxide anion [117]. In addition, the polymorphism GSTP1 rs1695 [118] and the combined GSTM1 and GSTT1 null polymorphisms [119] have been associated with melanoma susceptibility and with further increase in melanoma risk. These findings strongly indicate that oxidative stress is a driver of melanomagenesis [120].

## 7. Oxidative Stress Modulates DNA Methylation in Melanomagenesis

**Figure 2:**
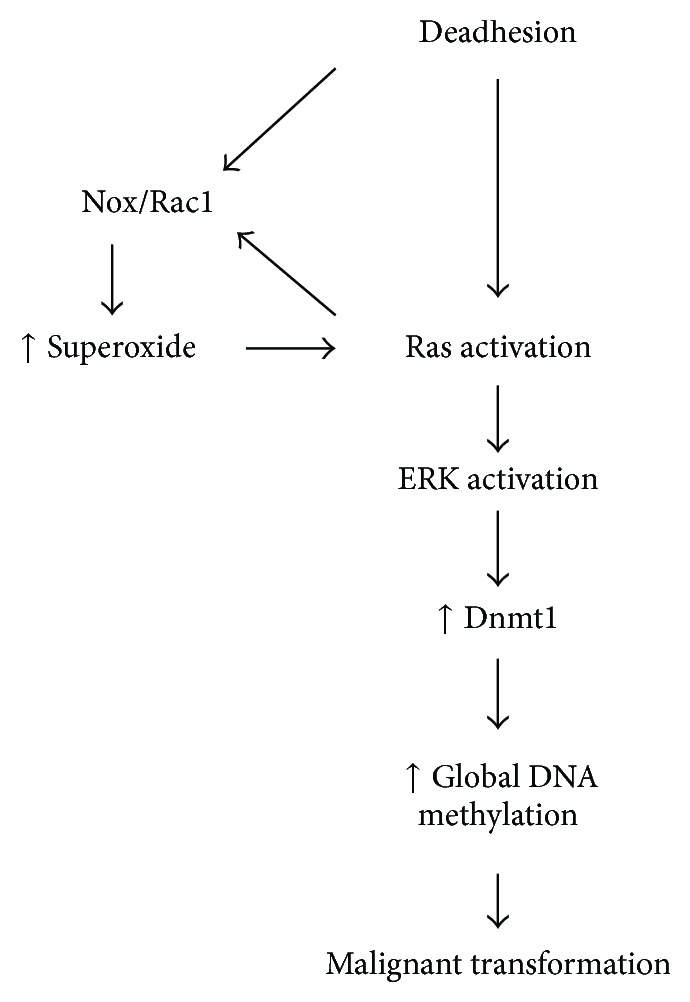
The effect of stress and its relationship with Ras/Rac1/MEK/ERK signaling pathway and epigenetic mechanisms regulation. Ras/Rac1/MEK/ERK signaling pathway seems to be regulated by and regulates superoxide anion production during melan-a anchorage blockade, and its activation could be responsible for the high Dnmt1 protein level and changes in global DNA methylation during the loss of cell adhesion. Anchorage blockade might promote epigenetic reprogramming in order to adapt the melanocytic cells to the new microenvironmental condition and contribute to the acquisition of a malignant phenotype (from [144]).

It was at first expected that aberrant DNA methylation would be infrequently implicated in melanomagenesis, as UV irradiation—that is deeply involved in melanoma—is believed to mainly cause gene mutations rather than epimutations. However, unexpectedly, epigenetic silencing of various tumour suppressor genes has been so far observed during melanoma development, progression, and metastasis [121–125]. Melanoma exhibits either global hypomethylation or local hypermethylation at the tumour suppressor gene level [126–128]. Nonetheless, the degree of global hypomethylation does not discriminate benign nevus from melanoma [129], as instead the survey on specific hypermethylation of tumor suppressor genes does [130, 131]. In this regard, we have shown that a high frequency of hypermethylation of* p16* ^*INK4A*^ [132],* DcR1*, and* DcR2* [133] promoters occurred in cutaneous as well as uveal melanoma. However, the search for specific sites of hypermethylation in melanoma also allowed us to identify a different susceptibility of uveal and cutaneous melanoma to the epigenetic effects of cadmium. In fact, we showed that cadmium exposure led to aberrant methylation and silencing of p16^INK4A^ in uveal melanoma, hypermethylation, and deregulation of caspase 8 in cutaneous melanoma cells [134]. Epigenetic processes, such as DNA promoter methylation and histone acetylation/deacetylation, were shown to be key cellular events during tumorigenesis [135, 136], and particularly in melanogenesis, since melanoma cells employ the epigenetic machinery to cope with adverse events and acquire resistance to chemotherapeutics [137]. This is particularly intriguing in view of the excellent response to treatment with epidrugs by patients with mucosal and ocular melanoma, which are the forms of melanoma more resistant to chemotherapy [138]. The combined therapy with the DNMT and HDAC inhibitors, decitabine and panobinostat, and the chemotherapeutic agent temozolomide has proven to be very effective. Moreover, a complete response was obtained in one patient affected by mucosal melanoma after only two cycles. This result is of particular relevance, since, as mentioned, mucosal melanomas harbor* KIT* mutations, are generally negative for* BRAF*, and metastasize more frequently than cutaneous melanomas. Therefore, further studies on epigenetic modifications that occur in mucosal melanomas and the possibility of reverting these changes with specific epidrugs become crucial, even if the rarity of these tumors can hinder studying this melanoma subtype. DNMT inhibition followed by HDAC inhibition, and targeting key epigenetic events, could turn on or off specific pathways that confer resistance to chemotherapy and apoptosis. Although oxidative/nitrosative stress and changes in DNA methylation were observed in many tumor types, few reports are available about the correlation between these events and melanomagenesis. Recently, methylated genes implicated in the response to oxidative damage have been associated with the risk of developing melanoma or dysplastic nevi [139], thus suggesting a link between oxidative imbalance and hypermethylation in melanoma. A previous study has shown that the blockade of a melanocyte cell line anchorage to extracellular matrix resulted in increased ROS and NO^•^ levels [140]. These alterations were accompanied by an increase in GSH and malondialdehyde (MDA) levels and methylated DNA content due to an upregulation of DNMT1 and DNMT3b expression. The NOS inhibitor N(G)-Nitro-L-arginine methyl ester (L-NAME) and *N*-acetyl-L-cysteine (NAC) abrogate either the DNA hypermethylation or the production of superoxide anion. Although increased ROS intracellular levels induced by anchorage blockade have been considered as mediators of anoikis of several cell types [141, 142], they have also been associated with protection from apoptosis [57, 143]. Therefore, it can be argued that the decision to turn on the pathways of survival or death can be determined by the levels of ERK or p53, respectively [140]. Of particular significance is the presence of relevant amounts of p53 only in premalignant and not in malignant melanoma cells [140]. The mechanisms by which the oxidative stress induced in premalignant melanocytes by deadhesion modulates DNA methylation pattern and induces cell transformation have been recently elucidated through elegant experiments performed by Molognoni et al. [144]. They were able to show that melanocyte deadhesion increases superoxide anion levels and DNMT1 production as well as global DNA hypermethylation. The increase in superoxide anion is caused by the activation of Rac1 and leads to the activation of Ras pathway, which in turn activates Rac1. DNMT1 upregulation, global DNA methylation, and malignant transformation are achieved by Ras-induced ERK activation. The sequence of events triggered by the deadhesion of the melanocytic line melan-a is described in Figure 2.

Taken together, these findings have delineated the ways by which the oxidative stress induced by disanchorage of melanocytes from extracellular matrix may modify the epigenetic machinery and lead to melanoma. Thus, the aberrant oxidative pathways associate with sustained levels of stress, which might or might not be related to UV exposure, and appear to contribute to the development of melanoma through epimutations.

## 8. Conclusions

Melanocytes are particularly susceptible to oxidative stress owing to the prooxidant state generated during melanin synthesis and to the intrinsic antioxidant defenses that may be shattered in pathologic conditions. Oxidative stress can disrupt the homeostasis of melanocytes, causing damage to DNA, protein, and cellular components. Altered ROS levels could also affect epigenetic mechanisms and promote alterations in gene expression, thus leading to severe impairment of cell survival and cancer development. Understanding the complexity of oxidative stress pathways regulating the production of pigmentation, melanocyte growth, and malignant transformation has great potential to define the plethora of clinically effective compounds and give enormous promise for patients affected by this disease. A combinatorial strategy of epigenetic therapy with agents able to prevent the production and chronic accumulation of ROS along with standard chemotherapeutic regimens may help in overriding the intrinsic melanoma resistance to current approaches of treatment and hindering its recurrence.
